# A Soft Multi-Axis High Force Range Magnetic Tactile Sensor for Force Feedback in Robotic Surgical Systems

**DOI:** 10.3390/s22093500

**Published:** 2022-05-04

**Authors:** Muhammad Rehan, Muhammad Mubasher Saleem, Mohsin Islam Tiwana, Rana Iqtidar Shakoor, Rebecca Cheung

**Affiliations:** 1 Department of Mechatronics Engineering, National University of Sciences and Technology, Islamabad 44000, Pakistan; mrehan.mts19ceme@mts.ceme.edu.pk (M.R.); mohsintiwana@ceme.nust.edu.pk (M.I.T.); 2 National Centre of Robotics and Automation (NCRA), Islamabad 44000, Pakistan; rana.iqtidar@mail.au.edu.pk; 3 Department of Mechatronics Engineering, Air University, Islamabad 44000, Pakistan; 4 Institute for Integrated Micro and Nano Systems, School of Engineering, University of Edinburgh, Scottish Microelectronics Centre, Edinburgh EH9 3FF, UK; r.cheung@ed.ac.uk

**Keywords:** multi-axis, magnetic tactile sensor, robotic surgery, force range, Hall sensor, elastomer

## Abstract

This paper presents a multi-axis low-cost soft magnetic tactile sensor with a high force range for force feedback in robotic surgical systems. The proposed sensor is designed to fully decouple the output response for normal, shear and angular forces. The proposed sensor is fabricated using rapid prototyping techniques and utilizes Neodymium magnets embedded in an elastomer over Hall sensors such that their displacement produces a voltage change that can be used to calculate the applied force. The initial spacing between the magnets and the Hall sensors is optimized to achieve a large displacement range using finite element method (FEM) simulations. The experimental characterization of the proposed sensor is performed for applied force in normal, shear and 45° angular direction. The force sensitivity of the proposed sensor in normal, shear and angular directions is 16 mV/N, 30 mV/N and 81 mV/N, respectively, with minimum mechanical crosstalk. The force range for the normal, shear and angular direction is obtained as 0–20 N, 0–3.5 N and 0–1.5 N, respectively. The proposed sensor shows a perfectly linear behavior and a low hysteresis error of 8.3%, making it suitable for tactile sensing and biomedical applications. The effect of the material properties of the elastomer on force ranges and sensitivity values of the proposed sensor is also discussed.

## 1. Introduction

There has been rapid advancement in the use of robotics in medical and healthcare applications in the past decade. To access the internal organ in a minimally invasive robotic surgery (MIRS), the surgeon uses small skin incisions using specially designed low-profile surgical instruments and flexible catheters [1]. As a result, the anesthesia time, incision size, blood loss during operation, chances of infection after the operation, trauma and hospitalization time are reduced [2]. One major limitation of the robotic surgical systems in the MIRS is that the surgeon has no idea of the interaction force of the surgical instrument with the body organ during the procedure. This may lead to excessive force exertion and damage to the body organ or tissue. Recent advancements in sensing technologies have made it possible to develop tactile force feedback sensors and install them on surgical instruments to detect the force of interaction of the instrument with the body organ.

Surgical instruments such as forceps and grippers have size constraints according to their jaws, therefore the sensor must be compliant with the size constraints. The sensing element must be of soft and biocompatible materials so that it should not damage any organ during the surgical operation. Soft materials such as plastic [3], silicone [4], yarn and fabrics [5] are being used in MIRS as sensing elements to make the sensors soft and able to interact with biological tissues. In the literature, different transduction mechanisms including capacitive [6,7,8], piezoresistive [9,10,11], piezoelectric [12,13], inductive [14,15], conductive rubbers [16,17] and optical [18,19] have been used for the development of tactile force sensors. The main limitations of these transduction mechanisms are nonlinearity, hysteresis, expensive fabrication and inconsistency in the output readings [20].

The tactile sensors based on the magnetic transduction mechanism offer the main advantages of linearity, low hysteresis, better repeatability, simple fabrication and physical robustness in comparison to other transduction mechanisms [21,22]. Youssefian et al. [23] proposed Hall sensors based magnetic tactile sensor for robotic systems with a maximum force range of 1.2 N in normal and 0.2 N in the shear direction. The authors used a hollow dome of elastomer, which was a limitation of the sensor as it made the sensor unable to withstand high loads. Jamone et al. [24] proposed a single axis force sensor for robotic fingers using a single Hall sensor with an embedded magnet in the elastomer. A normal force range of 0–3 N was reported, with a minimum measurable force of 0.01 N. The main limitations of this sensor were that it was not able to measure the shear forces and the nonlinear output response. Wang et al. [25] proposed a tri-axis magnetic tactile sensor, based on a 3D Hall sensor, which can measure both the normal and shear forces with a force range of 0–4 N and 0–1 N in the normal and shear axis. The reported sensor could not measure the angular force and differentiate between the shear force directions owing to the limitations of the commercially used tri-axis magnetometer. Chathuranga et al. [26] presented a design and magnetic field modelling of a tri-axis force sensor based on magnetic transduction mechanism using three single-axis Hall sensors with a normal and shear force range of 0–2 N and 0–1.6 N, respectively. Tomo et al. [27] proposed a tactile skin for robotic applications, based on the array of 3D Hall sensors, with a normal force measurement range of 0–14 N and 45° force range of 0–5 N. The main limitation of the reported magnetic sensing array was crosstalk between the sensing elements. A 4 -DOF load cell based on two tri-axis magnetometers which were able to detect forces in three axes and single-axis torque about the *z*-axis was reported in [28]; a high force range of up to 40 N in normal, 15 N in shear and torque up to 0.8 Nm about the *z*-axis was reported. The limitation of the sensor was its large size, because it was fabricated on the concept of a load cell. A soft distributed tri-axis force sensors based skin for robotic finger using 3D Hall sensors with a normal and shear input force sensing range of 0–6 N and 0–1 N, respectively, was presented in [29]. Kumar et al. [30] proposed a sensor system based on a single axis Hall sensor to assess the applied forces to an eye during ocular digital massage in an ophthalmic anesthesia training model. The sensor was capable of measuring forces up to 30 N in the normal direction, whereas the shear force data was not reported. Jones et al. [31] proposed a magnetic based tactile sensor for upper limb splinting to assess the pressures at contact points. The sensor was able to measure pressure up to 4 kPa in the shear direction and 30 kPa in the normal direction.

The decoupling of the force into its normal and shear components is an important criterion for force feedback in MIRS since it allows to achieve tissue characterization and grasping stability. The medical forces are generally in the range of ±10 N and maybe as high as 25 N for laparoscopic surgical tools [32]. Most of the tri-axis magnetic tactile sensors presented in the literature have a low force range. In this study, a low cost soft magnetic tactile sensor is proposed with a focus on the small size, high force range for both static and dynamic loading, low hysteresis error and high sensitivity. A solid elastomer is used in this study compared with the hollow elastomer approach-based designs in the literature to achieve a high loading range. The magnets are embedded inside the elastomer such that they are completely encased within the elastomer, and the proposed sensor remains soft and robust.

## 2. Proposed Magnetic Tactile Sensor

### 2.1. Sensor Design and Working Principle

**Figure 1:**
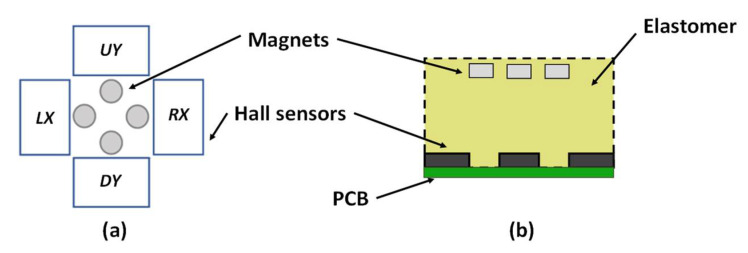
Schematic design of the proposed magnetic tactile sensor: (**a**) top view, (**b**) side view.

The schematic of the proposed magnetic tactile sensor is shown in Figure 1. The proposed design consists of a soft sensing element on a hard base. The sensor is made using flexible elastomers to make it suitable for tactile sensing applications in the field of robotic surgery and other industrial robotic applications. Four Honeywell SS39ET surface mounted device (SMD) Hall sensors are mounted on FR4 double layer printed circuit board (PCB). At the bottom of the sensor, there is a connector for powering up the sensor and data acquisition. There is a 10 mm layer of a soft elastomer on the top of the PCB with four grooves for embedding the cylindrical magnets. The four Neodymium N30H magnets with a height of 2 mm and a diameter of 3 mm are embedded in the elastomer. The initial gap between the embedded magnets and SMD Hall sensors is 6 mm. On top of the elastomer, there is a circular hard fiber sheet followed by another layer of elastomer. This hard fiber sheet allows a uniform displacement of magnets corresponding to an applied input force both in the normal and shear axis. The thickness of the sensing element consisting of elastomer with embedded magnets, a hard fiber sheet and the top flexible elastomer is 17 mm.

When a force is applied in the normal direction, all four magnets move downwards in the *z*-axis and the magnetic field strength increases across the SMD linear Hall sensors mounted on a dual-layer PCB. When a force is applied in the positive *x*-direction, the magnetic field strength of Hall sensor *RX* increases, while that for *LX* decreases and remains constant for the *UY* and *DY* Hall sensors. Similarly, for an input force in the positive *y*-direction, the magnetic field strength of *UY* increases and *DY* decreases and remains constant for the *LX* and *RX*. For an input force at an angle of 45°, the magnetic field strength in the Hall sensors *UY* and *RX* increases and for the *LX* and *DY*, decreases. Table 1 shows the complete sensing scheme for an input force in the normal, shear and angular directions.

### 2.2. Sensor Fabrication Process

**Figure 2:**
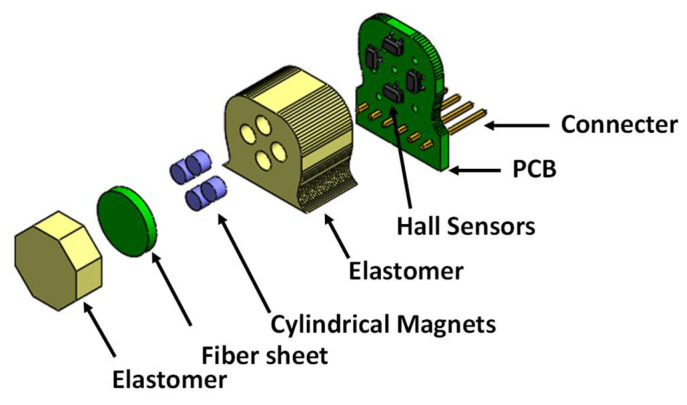
Exploded view of the proposed magnetic tactile sensor.

**Figure 3:**
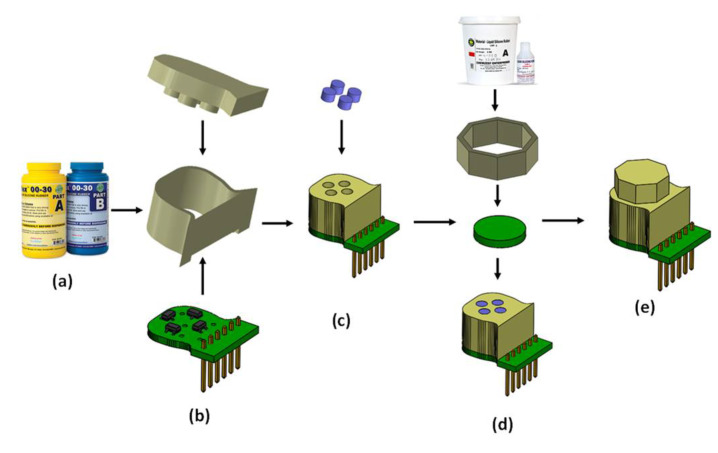
(**a**) Ecoflex 00-30 elastomer; (**b**) assembled PCB and 3D printed molds; (**c**) magnets and cured elastomer; (**d**) RTV-528 elastomer, 3D printed mold, fiber sheet and embedded magnets in elastomer; (**e**) final assembled sensor.

**Figure 4:**
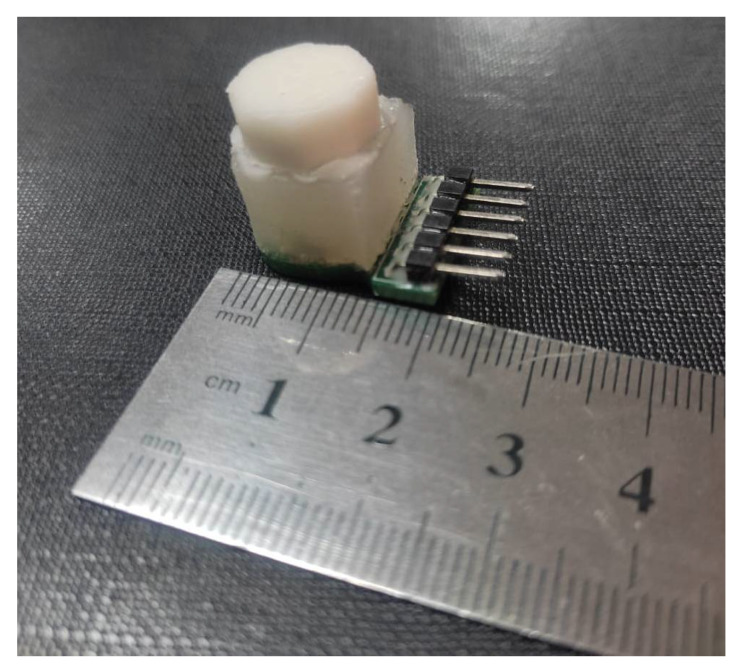
Fabricated sensor with soft material based octagonal-shaped dome for force application.

The exploded view of the proposed magnetic tactile sensor is shown in Figure 2. For the proposed magnetic tactile sensor, a hard double layer PCB acts as the base. The SMD Hall sensors are mounted on the PCB using surface mounted technology (SMT) soldering. There are two different kinds of elastomers used in the proposed sensor: liquid silicone rubber (Smooth-On Ecoflex 00-30 Inc., Macungie, PA, USA) and RTV-528 silicone rubber. The Ecoflex 00-30 is available in two parts, which are mixed in equal volumetric ratio for 3 min, degassed and poured into the molds. Similarly, RTV-528 is available along with its curing agent which is mixed with the liquid silicone rubber drop by drop using a syringe. Afterwards, the mixture is degassed and poured into the molds. The elastomers are shaped using 3D printed molds made of polylactic acid (.PLA). The Ecoflex 00-30 is cured for 4 h at room temperature and RTV-528 is cured for 24 h with 1% curing agent by volume at room temperature. The magnets are glued to the hard fiber sheet using cyanoacrylate glue. Ecoflex 00-30 is a softer elastomer in comparison with the RTV-528, hence RTV-528 is used as the top surface of the device so that the applied force is uniformly distributed. Figure 3 shows the different steps involved in the sensor fabrication and Figure 4 shows the final fabricated sensor. The total cost of the sensor components is not more than $15, thus making it a low-cost and disposable sensor, keeping in view the application of such disposable sensors in robotic surgical systems where sensors are to be replaced frequently such as in robot-assisted laparoscopic surgery [32]. Table 2 shows a brief part-by-part cost analysis for the proposed magnetic tactile sensor.

## 3. Sensor Modelling

### 3.1. FEM Modelling

**Figure 5:**
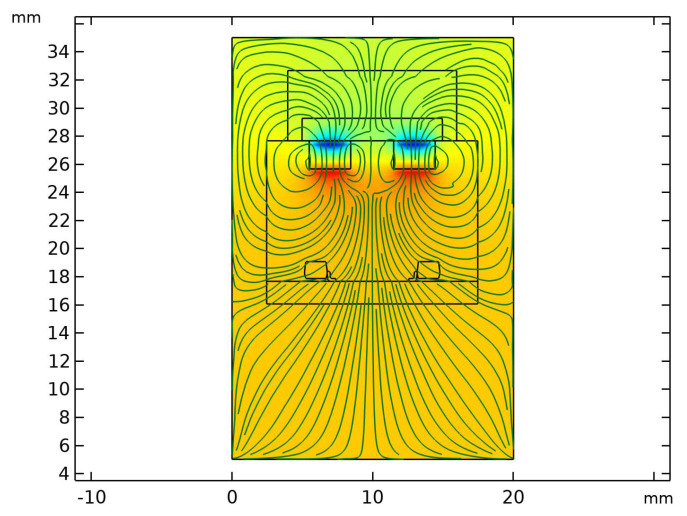
Cross-sectional view of the tactile sensor showing magnetic field lines originating from the magnets.

The magnetic field of magnets is modelled using the COMSOL Multiphysics finite element method (FEM) tool. The sensor is modelled considering a confined air space of size 20 mm × 25 mm × 30 mm. Figure 5 shows the magnetic field lines plot with the field lines originating from the magnets. The results show that the effective distance of the magnets from the Hall sensors is nearly 6 mm with a magnetic field strength of 0.00727 T.

**Figure 6:**
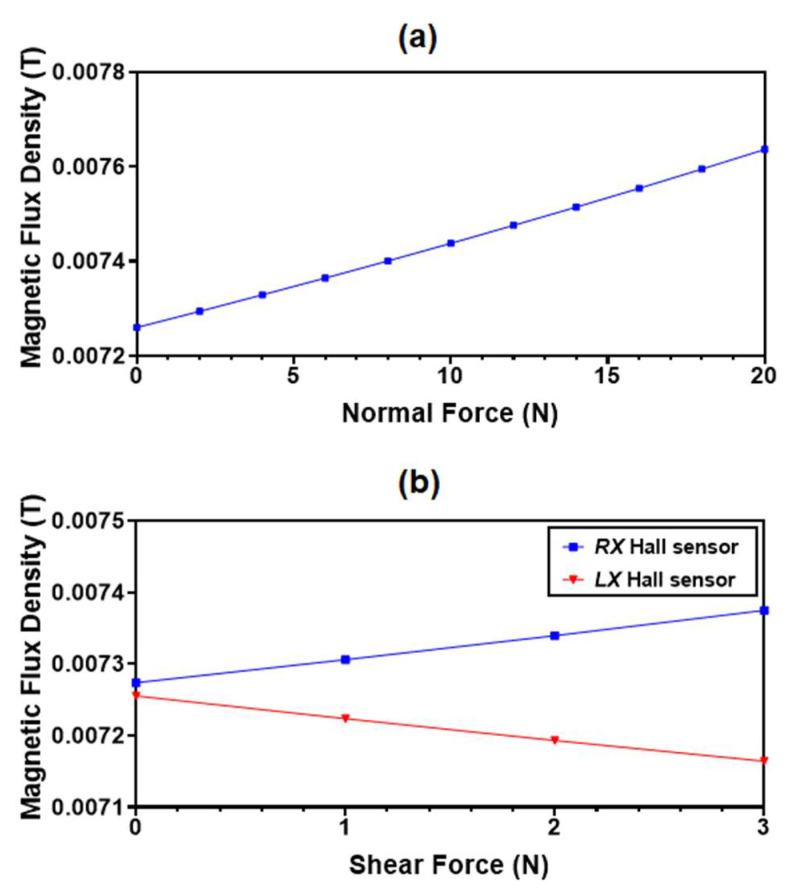
(**a**) Simulated average magnetic flux density value at all four Hall sensors for an applied normal force; (**b**) magnetic flux density value at RX and LX Hall sensors for applied shear force in the +x direction.

To analyze the effect of the normal and shear input force on the variation in the magnetic flux density on the Hall sensors, an analysis is performed using the COMSOL (solid mechanics module and magnetic field no currents module). The change in the magnetic flux density values with the application of an input normal force is shown in Figure 6a. The magnet flux density increases from 0.00726 T to 0.00764 T for an input force up to 20 N. Similarly, for a shear input force, the magnetic flux density in the *RX* Hall sensor increases from 0.00727 T to 0.00737 T and in the *LX* Hall sensor decreases from 0.00725 T to 0.0071 T as shown in Figure 6b.

**Figure 7:**
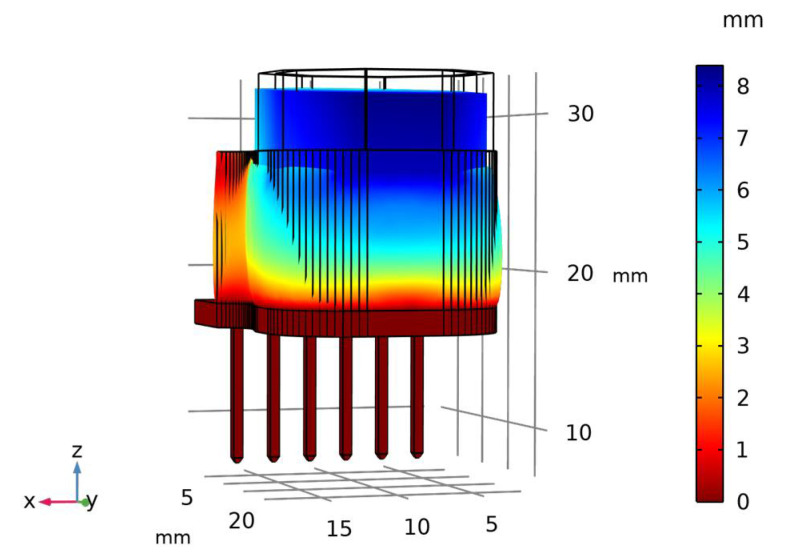
Displacement profile of the sensor at maximum input force of 20 N.

The displacement of the magnets embedded in the Ecoflex 00-30 elastomer is analyzed through the FEM analysis. The material properties assumed for the elastomer are: density of 1070 kg/m^3^, Young’s Modulus of 0.1 MPa and the Poisson ratio of 0.49 [33]. Figure 7 shows the displacement profile of the sensor in the normal direction at a maximum force of 20 N with a maximum displacement of 6 mm in the magnets, which is the maximum allowed displacement as per the design.

According to the FEM simulations, the initial value of the magnetic flux density at the Hall sensors is 0.00727 T. The output of the Hall sensors used in the sensor design saturates when the magnetic flux density is increased to 0.1 T. The maximum allowed displacement of magnets embedded in the elastomer is 6 mm and, at this, the magnetic flux density value at the Hall sensors is 0.00764 T. This value is much less than the saturation threshold value. This shows that the 6 mm gap between the Hall sensors and the magnets may be increased further to increase the force range, although this can lead to a lower sensitivity as well as undesired displacement in the shear direction.

### 3.2. Mathematical Modelling

The proposed magnetic tactile sensor is designed such that the inherent positions of the Hall sensors and the magnets allow the decoupling of input forces in normal, shear and angular directions. Equations (1)–(3) show the resultant voltage change due to an applied force in the normal and shear directions.

(1) $$\Delta V_{z}=\frac{\left(\Delta V_{UY}+\Delta V_{DY}+\Delta V_{LX}+\Delta V_{RX}\right)}{4}$$

(2) $$\Delta V_{x}=\Delta V_{RX}-\Delta V_{LX}$$

(3) $$\Delta V_{y}=\Delta V_{UY}-\Delta V_{DY}$$

where $\Delta V_{x}$, $\Delta V_{y}$ and $\Delta V_{z}$ are the resultant change in output voltage values of the Hall sensors for input forces in *x*, *y* and *z* directions, respectively. For a normal force, an average voltage change in all the four Hall sensors is used as the output voltage. For a shear input force, the differential output of two Hall sensors along the axis of applied force is used as an output.

As shown in Table 1, the proposed sensor is also able to detect input forces at different angles. To decouple the applied forces at different angles, mathematical equations are derived. The model works on the differential of the increase in value of the set of two sensors in the opposite quadrants. The voltage outputs *V_UY_*, *V_RX_*, *V_DY_* and *V_LX_* are from *UY*, *RX*, *DY* and *LX* Hall sensors, respectively. The resultant change in the output voltage of the sensor at different angles can be obtained as:

(4) $$\Delta V_{45{}^{\circ}}=(\Delta V_{UY}+\Delta V_{RX})-(\Delta V_{DY}+\Delta V_{LX})$$

(5) $$\Delta V_{135{}^{\circ}}=(\Delta V_{UY}+\Delta V_{LX})-(\Delta V_{DY}+\Delta V_{RX})$$

(6) $$\Delta V_{225{}^{\circ}}=(\Delta V_{DY}+\Delta V_{LX})-(\Delta V_{UY}+\Delta V_{RX})$$

(7) $$\Delta V_{315{}^{\circ}}=(\Delta V_{DY}+\Delta V_{RX})-(\Delta V_{UY}+\Delta V_{LX})$$

## 4. Experimental Validation

### 4.1. Experimental Setup

**Figure 8:**
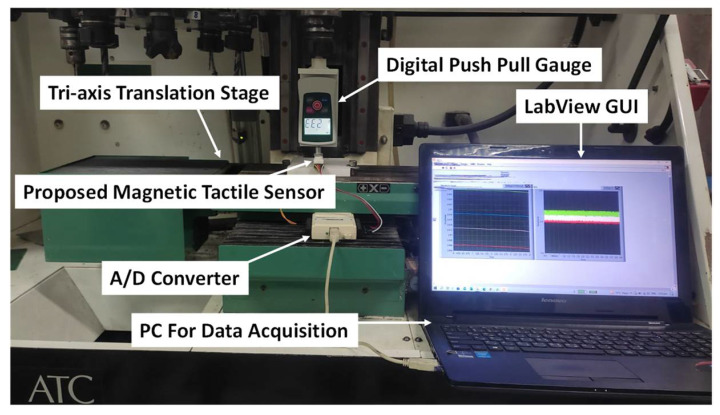
Experimental setup for the characterization of the magnetic tactile sensor.

The experimental setup used for the characterization of the magnetic tactile sensor is shown in Figure 8. The setup consists of a computerized numeric control (CNC) milling machine which is being used as a tri-axis translation stage. The sensor is mounted on the bed of the machine. A digital push–pull force gauge with a force range of 50 N and a resolution of 10 mN is mounted in the tool post. The minimum displacement step size of the machine is 1 µm. For static loading in normal and shear directions, the machine is controlled manually using an instrument panel and the force is recorded from the digital force gauge. For data acquisition from the proposed magnetic tactile sensor, a National Instruments USB-6009 DAC device is used in analog reading mode with National Instruments LabView software. The sampling rate of 2000 Hz with 400 samples is acquired and filtered using a 500 Hz low pass filter and moving average filter.

### 4.2. Testing and Results

**Figure 9:**
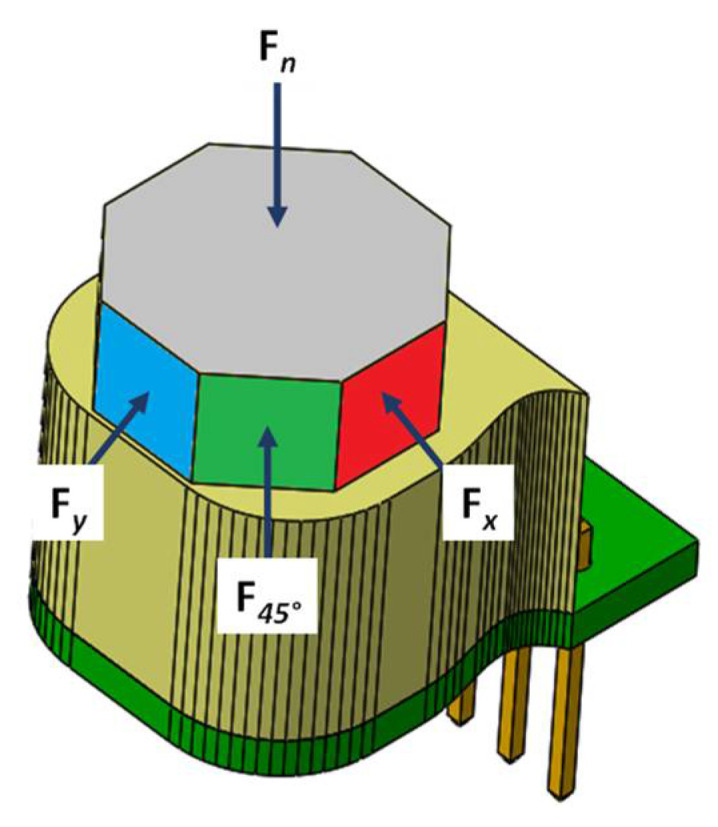
Applied force illustration using the octagonal dome.

**Figure 10:**
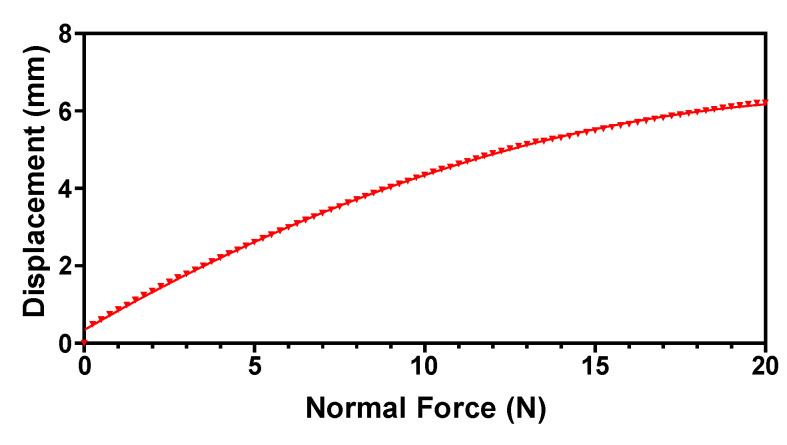
The displacement profile of the proposed tactile sensor for an input normal force range up to 20 N.

For the testing and validation of the magnetic tactile sensor, an octagonal-shaped dome is designed on top of the sensor such that forces in the normal, shear and angular directions can be applied. Figure 9 shows the dome with arrows showing the applied forces in different directions. A force in the normal direction is applied on the face, shown in grey color in Figure 9, with a step size of 0.1 N, while the sensor is stationary in the horizontal direction. A 5 s wait time is utilized to make the sensor values stable. Figure 10 shows the displacement profile of the elastomer with input force in the range of 0 to 20 N. The results show that the displacement in the elastomer increases linearly up to 5 N and there is a slight decrease in its value for force up to 20 N. This decrease in the deformation can be attributed to the hyperelastic material property of the elastomer.

**Figure 11:**
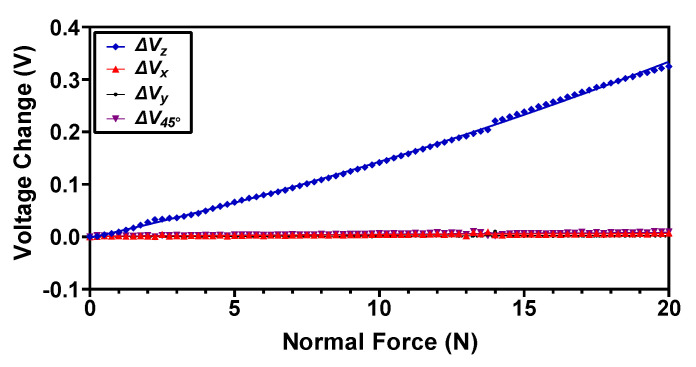
The output voltage response of the proposed magnetic tactile sensor for an applied normal force.

At a maximum applied force of 20 N, the displacement in the elastomer is 6 mm, which is the maximum gap between the magnets embedded in the elastomer and the Hall sensors. Figure 11 shows the output voltage change of the magnetic sensor obtained by measuring the individual change in voltage in the four Hall sensors and then averaging using Equation (1). The results show that the output response of the magnetic tactile sensor is relatively linear with a maximum change in the output voltage of 0.325 V at 20 N normal force. The voltage sensitivity of the sensor in the normal direction obtained from the experimental output voltage values is 16 mV/N. The output voltage change values for the shear axis and angular direction are negligible. This shows that the normal force response of the sensor is fully decoupled from the shear and angular force axis.

**Figure 12:**
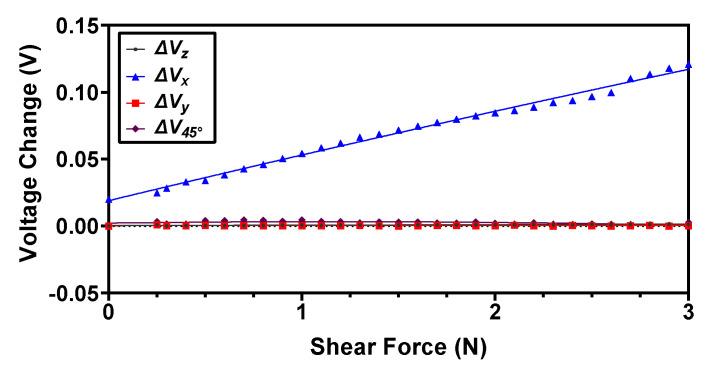
The output voltage response of the proposed magnetic tactile sensor for an applied shear force in the +x direction.

For the characterization of force range and sensitivity in the shear direction (*+x*), a force is applied with a step size of 0.1 N on the side of the dome face, which is shown in red color in Figure 9. For the applied force, the magnets displace in the *x*-axis and cause an increase in the magnetic flux density value of the *RX* Hall sensor and a decrease in the magnetic flux value of the *LX* Hall sensor. Thus, the output voltage of the *RX* Hall sensor increases with the increasing force, while that of the *LX* Hall sensor decreases. The difference of this change in voltage is calculated using Equation (2) and the resultant values are shown in Figure 12. It can be observed from the results that the voltage responses $V_{z}$, $V_{y}$ and $V_{45{}^{\circ}\;}$ remain constant for an applied force in the *+x* direction, thus depicting the decoupled behavior of the sensor in the case of an applied shear force. The recorded maximum force range in the shear direction is 3.5 N with a sensitivity of 30 mV/N. For an input force greater than 3.5 N, the voltage output from the Hall sensor saturates.

**Figure 13:**
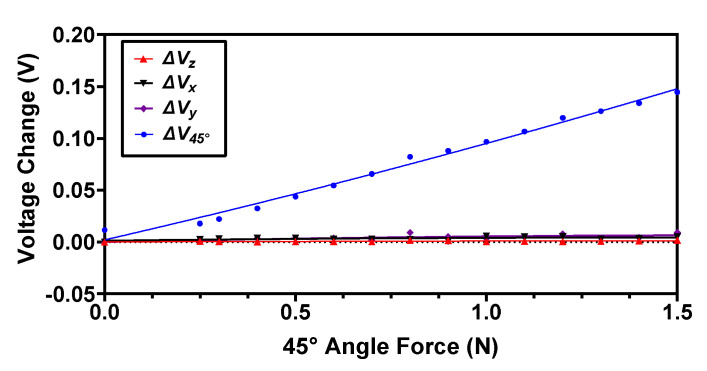
The output voltage response of the proposed magnetic tactile sensor for an applied force at an angle of 45°.

To analyze the response of the proposed magnetic tactile sensor, an angular input force at 45° is applied on the face shown in green color in Figure 9. For this force, the magnets are displaced such that the voltage output increases in the *RX* and *UY* Hall sensors, whereas it decreases for the *LX* and *DY*. The output voltage response *V_45°_* of this input force is calculated using Equation (4). The output voltage responses in normal and shear *x*, *y* denoted as *Vz*, *Vx* and *Vy,* respectively, are plotted as shown in Figure 13. The results show that the maximum input shear force at an angle of 45° that the proposed magnetic tactile sensor can detect is 1.5 N. The voltage sensitivity of the sensor for a 45° angular force is calculated to be 81 mV/N. Since the Hall sensors are arranged in a differential configuration for the shear axis *x* and *y*, the output voltage change using Equations (2) and (3) is negligible. Similarly, since for the normal force the output voltage change is obtained by averaging the output response of all four Hall sensors, the net change in voltage $\Delta V_{z}$ from Equation (1) is negligible for an applied input force at an angle of 45°. The results in Figure 11, Figure 12 and Figure 13 show that the proposed sensor design is capable of fully decoupling the normal, shear and angular forces applied to its surface.

**Figure 14:**
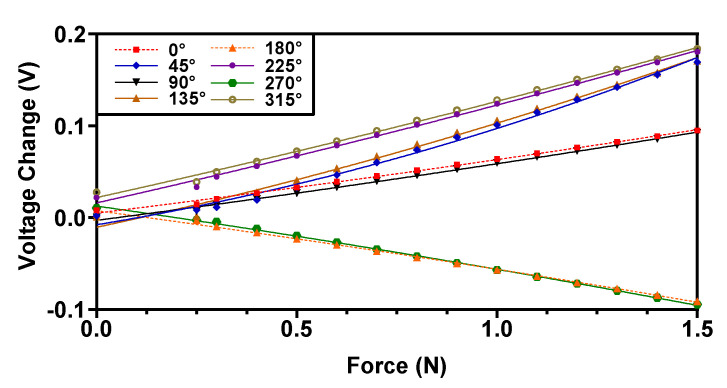
The output voltage response of the proposed magnetic tactile sensor for applied forces at different angles.

To assess the behavior of the proposed magnetic tactile sensor in the 3D space, the voltage response is recorded by applying angular forces at 0°, 45°, 90°, 135°, 180°, 225°, 270° and 315°. The recorded voltage response using the proposed mathematical model towards different angular forces is shown in Figure 14. The results in Figure 14 show that the proposed magnetic tactile sensor can sense angular forces in 3D space. There is a decreasing trend for the 180° and 270° angular forces due to Equations (2) and (3) that work on the principle of differential voltage changes. The voltage response due to these angular input forces is perfectly linear, and maximum voltage changes of 0.094 V, 0.092 V, −0.0911 V and −0.0944 V are recorded for angular forces at 0°, 90°, 180° and 270°, respectively. Similarly, for the angular forces at 45°, 135°, 225° and 315° maximum voltage changes of 0.169 V, 0.17 V, 0.18 V and 0.183 V, respectively, are recorded.

**Figure 15:**
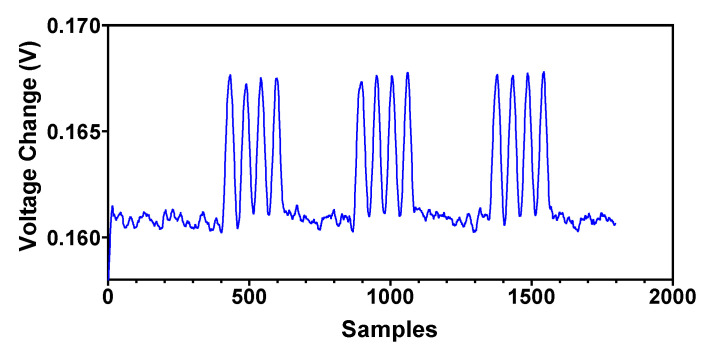
Dynamic response of the proposed magnetic tactile sensor for a force of 20 N at 4 Hz in the normal direction.

For measuring the dynamic contact force of the surgical instrument with body tissue or an organ it is very important to characterize the dynamic input force response of the tactile sensor. The type I and II fast-adapting mechanoreceptors in the human hand can detect vibrations in the frequency ranges of ~5 to 50 Hz and ~40 to 400 Hz, respectively. Thus, an ideal tactile sensor should be able to measure dynamic force above 400 Hz [34]. The grasping frequency of the surgical instrument in robotic surgery may vary depending upon the surgical procedure. For example, for robot assisted laparoscopic surgical systems a grasping frequency of 3 Hz is required [35]. Due to the limitations of the testing equipment, the magnetic tactile sensor presented in this paper is tested at a dynamic frequency of 4 Hz with a force magnitude of 20 N in the normal direction. The tri-axis translation stage is moved with a speed of 66 mm/s and the probe is incident on the face of the tactile sensor for 0.05 s. The response time of the loading and unloading forces is within 0.2 s. The response for the applied cyclic loading and repeatability of the sensor is shown in Figure 15. The results show that the sensor can detect dynamic force, and a voltage change of 0.160 V to 0.168 V can be seen upon loading and unloading with a 20 N force at 4 Hz repeatedly.

### 4.3. Force Calculation Based on the Experimental Data

**Figure 16:**
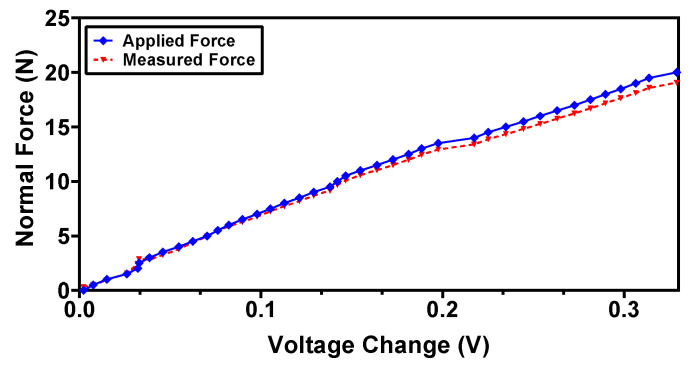
Measured vs. applied force in the normal direction using the back interpolation mathematical model.

**Figure 17:**
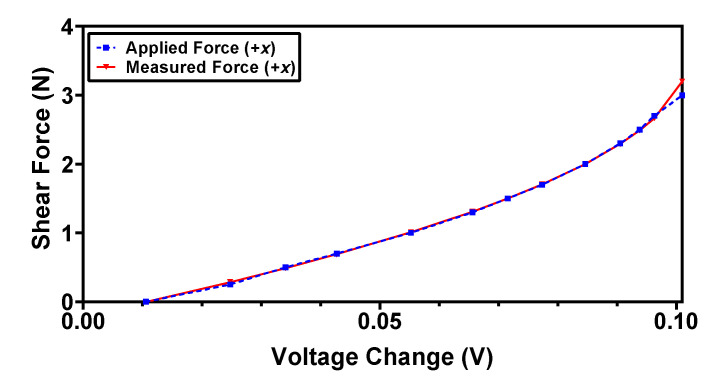
Measured vs. applied force in shear *+x*-direction using the back interpolation mathematical model.

To measure the forces in the robotic surgical applications the output of the sensor which is in the form of voltage needs to be converted to force. Hence, the proposed magnetic tactile sensor needs to be calibrated for a complete range of applied forces. For this purpose, a set of polynomial equations are derived by curve fitting a second-order polynomial expression on the output voltage data. From the recorded data the coefficients of polynomial expression are derived and the input forces are estimated using the coefficients of the polynomial expression in the quadratic formula with the measured voltage values. The second-order polynomial equations for the force estimation in the normal and shear axis are given as:

(8) $$\Delta Vz=0.0003F^{2}+0.011F+2.6989$$

(9) $$\Delta Vx=-0.0073F^{2}+0.0516F+0.0106$$

where Δ*V_z_* and Δ*V_x_* are the change in the output voltage of the sensor in the normal and shear axis, respectively, and *F* is the calculated applied force using the mathematical model. In order to validate the derived polynomial equations and to estimate the error between the measured and applied forces, the sensor is subjected to known input forces in normal and shear directions. Figure 16 and Figure 17 show the actual applied force and measured force (using Equations (8) and (9)) in the normal and shear axis, respectively. The results show that the measured forces are in close agreement with the actual applied forces with a maximum error of 4.9% and 6.2% for the normal and shear force, respectively.

## 5. Discussion

**Figure 18:**
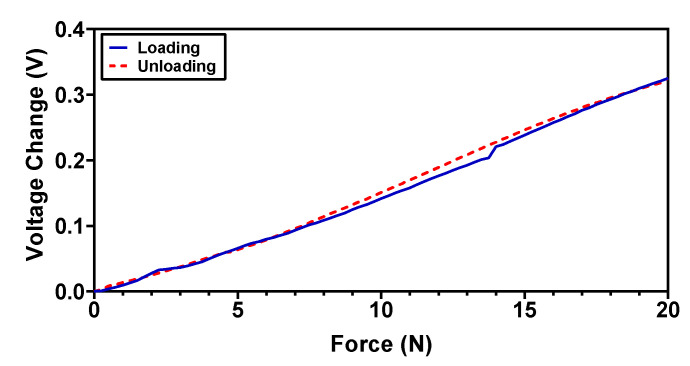
Hysteresis of the proposed magnetic tactile sensor in the normal direction.

For the applications where fast recovery time and dynamic force response is required, the tactile sensor’s hysteresis error should be minimum. For the proposed magnetic tactile sensor, the hysteresis error is characterized by a normal force loading–unloading cycle for a force range up to 20 N. The results in Figure 18 show that the sensor hysteresis error is relatively low and is 8.4% for the complete force range. The hysteresis error is dependent on the elastomer properties and in the case of polymer-based elastomers such as the Ecoflex 00-30 elastomer, the hysteresis error values are small, making them appropriate for applications where a fast dynamic response is needed [32].

**Figure 19:**
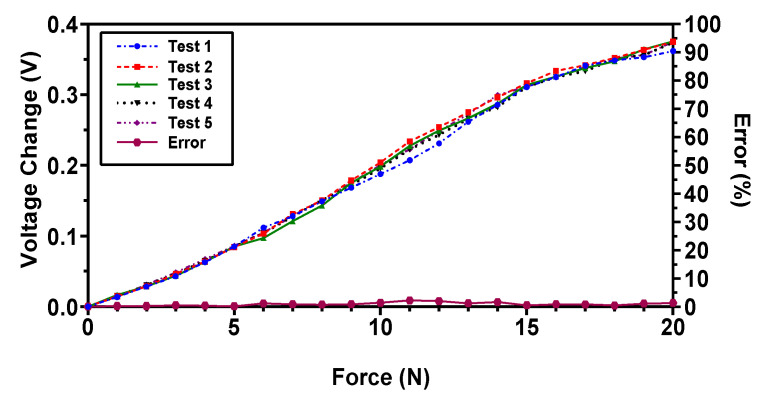
The voltage response of the proposed magnetic tactile sensor towards a force in the normal direction for five loading test cycles.

The repeatability of the sensor is an important criterion for tactile sensors used in robotic surgical systems and biomedical applications [36] to achieve better output reading consistency. In order to assess the repeatability of the proposed magnetic tactile sensor, the sensor is subjected to repeated loading for five loading cycles with different time delays. The voltage response of the sensor is recorded for five different loading cycles with a maximum force range of 20 N in the normal direction as shown in Figure 19. The sensor depicts good repeatability with a maximum relative standard deviation error of 6.4%.

Most of the magnetic tactile sensor designs reported in the literature use 3D Hall sensors for measuring multi-axis forces, but this makes their cost high as these Hall sensors are much more expensive than the ones used in the proposed tactile sensor design. Additionally, the 3D Hall sensors require complex interfacing and signal filtering to achieve smooth outputs. Table 3 shows the comparison between the proposed magnetic tactile sensor with the magnetic tactile sensors reported in the literature. It shows that the designs proposed in this work have a higher force range and resolution as well as a low hysteresis value, whereas the size of the proposed magnetic tactile sensor is comparable to the designs proposed in the literature. Moreover, the proposed magnetic tactile sensors are also capable of measuring angular forces.

**Figure 20:**
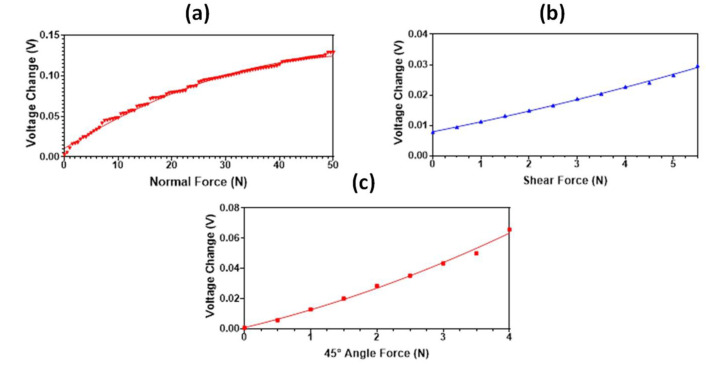
The output voltage response of the proposed magnetic tactile sensor with RTV-528 elastomer: (**a**) normal force, (**b**) shear force in *+x* direction, (**c**) 45° angular force.

In addition to better range and low hysteresis values, the proposed magnetic tactile sensor also has a robust design as the fragile elements (Hall sensors) do not directly come in contact with the applied force. This makes them less susceptible to damage from excessive loading as the force is transmitted through the elastomer. Another benefit of the magnetic tactile sensor design is the absence of electrical connections in the top layer; hence, the sensor can deform upon force application without damaging any fragile wires. Due to the modular design, some components of the sensor such as the elastomer can be replaced easily when different force ranges and sensitivity values are required. The proposed sensor is easy to repair and, due to its inexpensive fabrication as well as the low cost of the components, it can be referred to as a disposable sensor. The modular design of the sensor allows for easy replacement of the elastomer, therefore the proposed magnetic tactile sensor was also tested with RTV-528 silicone rubber as an elastomer. The sensitivity and force range of the sensor is dependent upon the stiffness of the elastomer. The Ecoflex 00-30 is a relatively soft elastomer as compared with RTV-528 silicone rubber; its Young’s modulus value is 0.454 MPa, whereas for Ecoflex 00-30 it is 0.125 MPa. Due to the high Young’s modulus value of RTV-528 in comparison with Ecoflex 00-30, a better force range up to 50 N, 5.5 N and 4 N is achieved for the sensor in the normal, shear and 45° applied force, respectively, as shown in Figure 20. However, the calculated force sensitivity values in the normal, shear and 45° angle directions are 2.52 mV/N, 3.4 mV/N and 25 mV/N, respectively, which are much less than the sensor with Ecoflex 00-30 as an elastomer. Table 3 shows the comparison of the proposed magnetic tactile sensor, using Ecoflex 00-30 as an elastomer, with the magnetic tactile sensors presented in the literature. The comparison shows the proposed magnetic sensors allow to achieve a higher force range and can decouple normal, shear and angular applied force, which is required for the robotic surgical systems.

The practical aspects of the proposed magnetic tactile sensor include the palpation probes and portable pen like devices for tumor stiffness detection and oral cancer screening. The proposed magnetic tactile sensor can be mounted on the needle block devices in ophthalmic anesthesia training models and simulators [30], where the force applied to the eyeball during ocular digital massage needs to be measured. Moreover, the proposed sensor can be integrated with surgical grippers and graspers used in robotic surgical systems and MIRS to assess the applied force and its direction, which can lead to better grasping stability and fewer chances of damage to tissues and organs.

One promising way to improve situational awareness of the robotic surgical systems and surgeons during surgical procedures is to incorporate haptic feedback devices with the proposed magnetic tactile sensor. The future prospect of this study is to propose a tactile sensing feedback mechanism for robotic surgical systems; this can be achieved using micro-actuators and dielectric soft elastomer actuators. The main aim is to propose a surgical pen like device that comprises the proposed magnetic tactile sensor and a haptic feedback device to stimulate the finger of the surgeons during any surgical procedure and needle block functions for ophthalmic anesthesia and ocular digital massage.

## 6. Conclusions

In this work, the design and experimental characterization of a mesoscale multi-axis magnetic tactile sensor is presented. The proposed sensor is capable of measuring normal, shear and angular forces. A higher force range of 0–20 N is reported in the normal direction, which shows that the sensor can be used for force feedback in a robotic surgical system. In the shear and angular direction, the experimentally demonstrated force range is 0–3 N and 0–1.5 N. A maximum hysteresis error of 8.4% for loading and unloading cycles and a relative standard deviation error of 6.4% is recorded for the repeatability tests. The design of the sensor includes an inherent decoupling of the applied forces such that there is no crosstalk between the normal, shear and angular forces. The sensing scheme and corresponding developed mathematical model allows approximating the input force in different axis efficiently. The sensor is fabricated using conventional manufacturing techniques, including 3D printing with low-cost components, which makes the proposed sensor a low-cost disposable sensor. To avoid damage to the organ or body tissues in robotic surgery, the top elastomer surface of the magnetic tactical sensor is fabricated using a soft material and, to achieve robustness, the magnets are embedded inside the elastomer.

## Figures and Tables

**Table 1:** Look-up table for the magnetic field strength against applied force.

Force Direction	Magnetic Field Strength			
	*B-_RX_*	*B-_UY_*	*B-_DY_*	*B-_LX_*
Normal (*z*)	↑	↑	↑	↑
Shear (*+x*)	↑	■	■	↓
Shear (−*x*)	↓	■	■	↑
Shear (*+y*)	■	↑	↓	■
Shear (−*y*)	■	↓	↑	■
Angle (45°)	↑	↑	↓	↓
Angle (135°)	↓	↑	↓	↑
Angle (225°)	*↓*	*↓*	↑	↑
Angle (315°)	↑	*↓*	↑	*↓*

**Table 2:** Cost of each component for proposed magnetic tactile sensor.

Parts	Cost
PCB	$2
SMD Hall sensors	$6
3D printed moulds	$2
Elastomer	$3
Magnets	$2

**Table 3:** Performance parameters of the proposed sensor in comparison with magnetic tactile sensors presented in the literature.

	Size	Elastomer	Force Range	Sensitivity	Resolution	Hysteresis
Youssefian et al. [23]	-	Silicone Rubber	(0–1.2 N) Normal force (0–0.2 N) Shear force	-	-	-
Jamone et al. [24]	-	Sylgard 186	(0–3 N) Normal force	0.2 V/N	0.01 N	-
Wang et al. [25]	Diameter: 12 mm Thickness: 12 mm	Ecoflex 00-30	(0–4 N) Normal force (0–1 N) Shear force	-	1.42 mN	3.4%
Chathuranga et al. [26]	Diameter: 15 mm	Dragon Skin 30	(0–2 N) Normal force (0–1.6 N) Shear force	-	-	10%
Tomo et al. [27]	Length: 20 mm Width: 23 mm	Ecoflex 00-30	(0–14 N) Normal force (0–5 N) 45° Shear force	-	-	-
Kumar et al. [30]	Diameter: 30 mm	-	(0–30 N) Normal force	1mV/mN	9.8 mN	-
Sensor presented in this work	Diameter: 15 mm Thickness: 17 mm	Ecoflex 00-30	(0–20 N) Normal force (0–3 N) Shear force (0–1.5 N) Angular force	Normal force: 16 mV/N Shear force: 30 mV/N Angular force: 81 mV/N	5 mN	8.4%

## Data Availability

Not applicable.
